# Case Report: Unexplained Mild Hypophosphatemia and Very High Serum FGF23 Concentrations

**DOI:** 10.1002/jbm4.10790

**Published:** 2023-08-09

**Authors:** Ariadne Bosman, Danielle MA Ratsma, Bram CJ van der Eerden, M Carola Zillikens

**Affiliations:** ^1^ Department of Internal Medicine Erasmus MC, University Medical Center Rotterdam The Netherlands

**Keywords:** HYPOPHOSPHATEMIA, IRON, IRON DEFICIENCY, FGF23, PHOSPHATE

## Abstract

Fibroblast growth factor (FGF)23 is one of the major regulators of phosphate homeostasis. Hypophosphatemia can lead to muscle weakness, fatigue, and osteomalacia. In the setting of hypophosphatemia, serum FGF23 can be measured to differentiate between FGF23‐mediated and non‐FGF23‐mediated renal phosphate wasting. C‐terminal FGF23 (cFGF23) assays detect both cFGF23 and intact FGF23 (iFGF23). Circulating FGF23 is regulated by 1.25‐dihydroxy‐vitamin D, parathyroid hormone (PTH), serum phosphate, and serum calcium but also by, for example, iron status, inflammation, erythropoietin, and hypoxia‐inducible‐factor‐1‐α. We present the case of a 48‐year‐old woman with unexplained mild hypophosphatemia, very high cFGF23, and normal iFGF23. The patient proved to have an iron deficiency. Iron deficiency alters the iFGF23‐to‐cFGF23 ratio. After initiation of iron treatment, cFGF23 strongly decreased. This case report illustrates the limitation of cFGF23 assays and urges clinicians to be aware that cFGF23 concentrations do not necessarily reflect iFGF23 concentrations and that alternative causes for its elevation should be considered (eg, iron deficiency). © 2023 The Authors. *JBMR Plus* published by Wiley Periodicals LLC. on behalf of American Society for Bone and Mineral Research.

## Graphical abstract

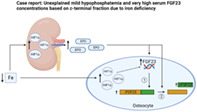

## Introduction

Inorganic phosphate is important for several metabolic processes, including intracellular signal transduction and energy production, and for mineralization of the skeleton by formation of hydroxyapatite. Acute severe hypophosphatemia can lead to neurological symptoms and impaired cardiac and respiratory function. Chronic hypophosphatemia is usually characterized by muscle weakness, fatigue, and the development of osteomalacia, accompanied by bone pain.^(^ ^1^ ^)^ One of the major regulators of phosphate is fibroblast growth factor (FGF)23.^(^ ^2^ ^)^ Recently it has been shown that FGF23 also interacts with iron metabolism and erythropoiesis.^(^ ^3^ ^)^ The current case illustrates the interaction between FGF23 and iron status, and challenges the applicability of C‐terminal FGF23 (cFGF23) assays for the work‐up of hypophosphatemia in the setting of FGF23‐related disturbances.

## Case Description

A 48‐year‐old woman was referred to the nephrologist of Erasmus Medical Center in 2012 suffering from bone and joint pain, fatigue, and muscle weakness for 6 months. Her family history was negative for bone‐related conditions and her physical examination did not show signs of growth retardation or bone deformities. Her medical history included asthma; microcytic hematuria; iron deficiency, for which she had used iron supplementation in the past; and pangastritis in 2010. She used salbutamol and formoterol/beclometasone for her asthma, and omeprazole. At biochemical evaluation, kidney function, serum sodium, potassium, and magnesium were within the reference range, but her phosphate was low, 0.71 mmol/L (normal range, 0.80–1.40). Albumin‐adjusted calcium was 2.25 mmol/L (normal range, 2.20–2.65), 25‐hydroxy‐vitamin D (25OHD) was 107 nmol/L (normal range, 50–120), 1.25‐dihydroxy‐vitamin D (1.25OH_2_D) was slightly increased, 197 pmol/L (normal range, 38–183), and intact parathyroid hormone (PTH) was 7.2 pmol/L (normal range, 1.4–7.3) (Table 1). The ratio of tubular maximum reabsorption of phosphate to glomerular filtration rate (TMP/GFR) was 0.57 mmol/L (normal range, 0.84–1.23) and cFGF23 (Immutopics, San Clemente, CA, USA^(^ ^4^ ^)^) was 234 RU/mL (normal range, <125), suggesting FGF23‐related renal phosphate wasting. There were no signs of a generalized tubulopathy, for example, glucosuria or proteinuria, and renal ultrasound showed no nephrocalcinosis or nephrolithiasis. Whole‐exome sequencing was performed for renal hypophosphatemic disorders and showed no mutations in genes *SLC34A1*, *SLC34A3*, or *SLC9A3R1*. She was treated with oral phosphate supplementation three times daily 20 mmol, resulting in repeatedly normal serum phosphate measurements, ranging from 0.86 to 1.39 mmol/L, and she was discharged from follow‐up.

**Table 1:** Laboratory Results During Follow‐up of the Patient

Serum	2012	2018	2019	2022 (before iron supplementation)	2022 (after iron supplementation)
Phosphate (mmol/L) (normal, 0.80–1.40)	0.71	0.77	0.83	0.77	0.95
25(OH)D (nmol/L) (normal, 50–120)	107	79	20	80	79
1.25(OH)_2_D (pmol/L) (normal, 38–183)	197	96.5	‐	‐	‐
Alkaline phosphate (U/L) (normal, <98)	‐	‐	177	93	92
PTH (pmol/L) (normal 0.68–4.40)	7.2^a^	3.9	3.3	‐	‐
cFGF23 (RU/mL) (normal <125)	234	697	935	‐	158
cFGF23 (RU/mL)^b^	‐	‐	‐	1260	‐
iFGF23 (pg/mL)^b^	‐	‐	‐	36	‐
Hemoglobin (mmol/L) (normal, 7.5–9.5)	8.2	‐	7.9	8.0	9.4
MCV (fl) (normal, 80–100)	81	‐	78	80	92
Iron (μmol/L) (normal, 10–30)	‐	‐	‐	5.7	18.5
Ferritin (μg/L) (normal, 10–140)	‐	‐	‐	6	67

Abbreviations: cFGF23 = C‐terminal fibroblast growth factor 23; iFGF23 = intact FGF23; MCV = mean corpuscular volume; PTH = parathyroid hormone.

^a^ Normal range PTH in 2012: 1.4–7.3 pmol/L.

^b^ C‐terminal FGF23 and iFGF23 were measured simultaneously in our research lab in 2022.

Six years later, in 2018, she was referred to the Bone Center of our hospital with persistent incapacitating fatigue and bone, muscle, and joint pain. She had never suffered a fracture. Serum phosphate was 1.39 mmol/L while on phosphate supplementation, but after cessation serum phosphate decreased to 0.77 mmol/L. At this time, albumin‐adjusted calcium was 2.45 mmol/L (normal range, 2.20–2.65), 25OHD was 79 nmol/L, 1.25(OH)_2_D was 96.5 pmol/L, and intact PTH was 3.9 pmol/L (normal range, 0.68–4.40). Iron status was not measured at this time but was normal earlier that year, when serum phosphate was 0.85 mmol/L. Twenty‐four‐hour urine calcium was 6.72 mmol (normal range, 2.5–7.5 mmol/24 h). Celiac disease was excluded based on absence of endomysial antibodies. Despite the fact that hypophosphatemia was mild and responded well to supplementation, TMP/GFR was repeatedly decreased, suggesting renal phosphate wasting. cFGF23, measured later that year, was 697 RU/mL (Table 1). The bone mineral density (BMD) assessment by dual‐energy X‐ray absorptiometry (DXA) revealed a *T*‐score of −2.1 SD at the femoral neck and −2.1 SD at the lumbar spine (normal, *T*‐score > −1 SD). Whole‐exome sequencing was repeated and showed no mutations in *FGF23* and *PHEX*. Our differential diagnosis included tumor‐induced osteomalacia (TIO) but physical examination and CT, FDG‐PET‐CT, and 68‐Gallium‐DOTATE PET‐CT did not show signs of a causative tumor.

In 2019, cFGF23 had increased up to 935 RU/mL, whereas serum phosphate was normal or slightly decreased without supplementation. At that time, serum 25OHD was decreased (20 nmol/L) and serum alkaline phosphatase (AF) was elevated, 177 U/L (normal range, <98) as well as bone specific alkaline phosphatase, 48.9 μg/L (normal range, <14.3). A bone biopsy showed severe osteoporosis and low bone turnover but no signs of osteomalacia. We initiated cholecalciferol after which serum 25OHD and AF normalized, but serum phosphate was still decreased, 0.77 mmol/L.

In the years that followed, cFGF23 concentrations kept rising and there was no finding of TIO on imaging. Because iron deficiency can be associated with hypophosphatemia and the patient had been treated with iron supplementation for iron deficiency in the past, we measured her iron status again in 2022. At this time, her hemoglobin was 8.0 mmol/L (normal range, 7.5–9.5), mean corpuscular volume was 80 fl (normal range, 80–100), but her iron was 5.7 μmol/L (normal range, 10–30), indicating iron deficiency. Because of the discrepancy between the very high cFGF23 and normal or slightly decreased serum phosphate, we decided to measure the C‐terminal and intact FGF23 (iFGF23) in our patient and in a healthy control in our research lab for calcium and bone metabolism because measurement of iFGF23 for clinical purposes was not available to us at that time. cFGF23 was 1260 RU/mL in the patient and 51 RU/mL in the healthy control (MicroVue Human FGF‐23 [C‐Term] EIA, Quidel, San Diego, CA, USA). iFGF23 was 36 pg/mL in our patient and 47 pg/mL in the healthy control (MicroVue Bone Human FGF‐23 [Intact] EIA, Quidel). After excluding gastrointestinal blood loss by performing gastroscopy and colonoscopy, we treated the iron deficiency with ferrous fumarate 200 mg three times daily. Serum iron increased to 18.5 μmol/L, serum phosphate was normal at 0.95 mmol/L without phosphate supplementation, and cFGF23 decreased from 935 to 158 RU/mL. The patient was pleased that the cause for the high cFGF23 concentrations was found and that she did not have a tumor. The iron supplementation improved her fatigue but the muscle pain remained.

## Discussion

We describe the medical history of a 48‐year‐old female patient with bone pain, fatigue, muscle complaints, mild hypophosphatemia, and very high cFGF23 concentrations. Because cFGF23 concentrations kept rising, we conducted extensive diagnostic procedures, including advanced imaging, in search of a growing tumor causing TIO, and genetic testing.^(^ ^5^ ^)^ It turned out she had normal iFGF23 concentrations and increased cFGF23 due to iron deficiency. This case raises two questions: (i) Can elevated cFGF23 cause mild hypophosphatemia in the setting of iron deficiency; (ii) Is the use of cFGF23 assays to differentiate between the causes of hypophosphatemia appropriate in the setting of serum FGF23 disturbances such as iron deficiency.

Serum phosphate is mainly regulated by 1.25(OH)_2_D, PTH, and FGF23. 1.25(OH)_2_D increases phosphate absorption from the intestine, whereas PTH and FGF23 stimulate renal phosphate excretion. Both 1.25(OH)_2_D and PTH induce bone resorption, which leads to release of phosphate. FGF23 also inhibits 1α‐hydroxylase, thereby decreasing the synthesis of 1.25(OH)_2_D.^(^ ^2^ ^)^ The differential diagnosis of hypophosphatemia can be divided into three major groups: hypophosphatemia from increased renal excretion, from decreased intestinal absorption, or due to intracellular shift of phosphate. Increased renal excretion can be subdivided into FGF23‐mediated and non‐FGF23‐mediated renal phosphate wasting.^(^ ^1^, ^6^ ^)^ Increased FGF23 concentrations can be caused by monogenetic disorders such as X‐linked hypophosphatemia or by a FGF23‐producing tumor in the setting of TIO.^(^ ^1^, ^5^ ^)^

FGF23 is mainly produced by osteoblasts and osteocytes. Classical regulators of circulating FGF23 include 1.25(OH)_2_D, PTH, alpha‐klotho, serum phosphate, and serum calcium. Non‐classical regulators of FGF23 include iron status, inflammation, erythropoietin, hypoxia‐inducible factor 1α (HIF1α), insulin and diabetes, and leptin.^(^ ^7^ ^)^ The *FGF23* gene encodes for a 251‐amino‐acid‐long glycoprotein that requires posttranslational O‐glycosylation in order to be stabilized and phosphorylation to be cleaved.^(^ ^3^, ^7^ ^)^ Currently, two types of FGF23 assays exist for the quantification of circulating FGF23 concentrations in humans. The first one is the C‐terminal assay, which detects both the active full‐length FGF23 and the C‐terminal fragments that are released after cleavage, by using antibodies that target epitopes in the C terminus. The second one is the intact FGF23 assay, which only detects the presumed active full‐length FGF23.^(^ ^3^ ^)^ In the Netherlands, FGF23 concentrations are determined at the Amsterdam University Medical Center by a C‐terminal FGF23 (cFGF23) assay (Immutopics) (upper limit of normal, 125 RU/mL).^(^ ^4^ ^)^

Edmonston and colleagues describe the application of an intact FGF23‐to‐C‐terminal FGF23 ratio (i:cFGF23), in combination with the concomitant serum phosphate level, in discriminating between different FGF23‐mediated syndromes.^(^ ^3^ ^)^ Two types of conditions that can lead to high cFGF23 with normal or low iFGF23 concentrations, resulting in a low i:cFGF23 ratio, have been described. On the one hand, there are conditions, including iron deficiency, inflammation, and increased erythropoietin (EPO), that stimulate *FGF23* transcription and cleavage, resulting in high cFGF23 but normal iFGF23 concentrations. On the other hand, there is a condition named tumoural calcinosis, which can be caused by a mutation in N‐acetylgalactosaminyltransferase 3 (*GALNT3)*, resulting in increased cleavage of iFGF23 with high cFGF23 and low iFGF23 concentrations.

The interaction between FGF23 and iron status and erythropoiesis has been extensively studied. Induction of iron deficiency by a low‐iron diet in wild‐type mice resulted in increased serum intact FGF23 and C‐terminal FGF23 concentrations, with a corresponding decrease in 1.25(OH)_2_D concentrations and an increase in urinary phosphate excretion but no change in serum phosphate.^(^ ^8^ ^)^ The role for iron in FGF23 regulation in humans was illustrated by Imel and colleagues in patients with autosomal dominant hypophosphatemic rickets (ADHR), which is caused by mutations in the *FGF23* gene, leading to impaired cleavage of iFGF23.^(^ ^9^ ^)^ In this study, low serum iron was associated with both elevated cFGF23 and iFGF23 in patients with ADHR, but only with elevated cFGF23 in healthy controls. This suggested that iron deficiency leads to increased *FGF23* expression, but increased cleavage retains homeostasis in humans without ADHR. Similarly, serum iron was found to be associated only with increased cFGF23 and not with iFGF23 in a study in healthy premenopausal women. In this study, serum phosphate correlated with iFGF23 in all women but with cFGF23 in black women only.^(^ ^10^ ^)^ In contrast, in a Swedish population‐based cohort study of elderly men, low serum iron was found to be associated with high serum iFGF23.^(^ ^11^ ^)^

**Fig. 1:**
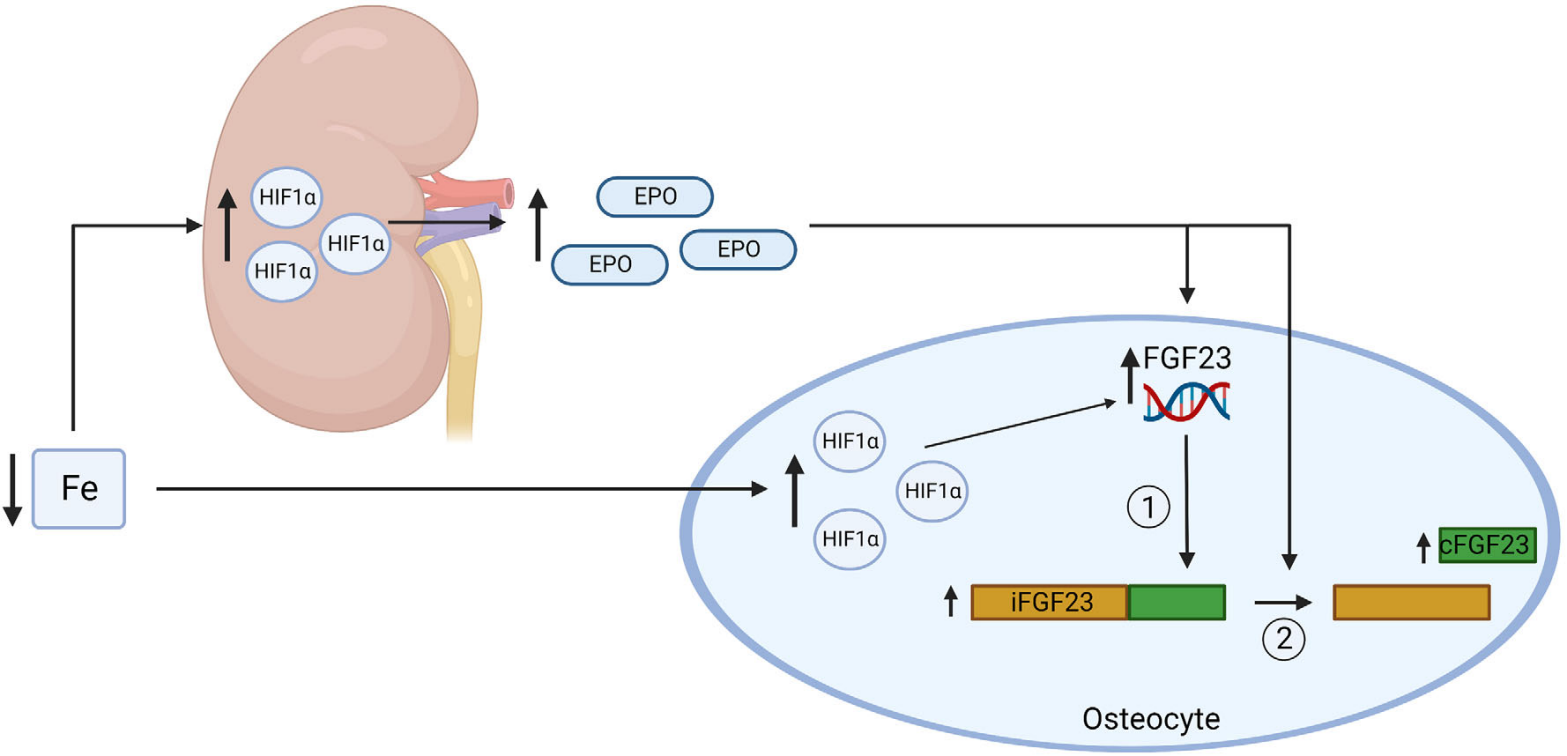
Potential mechanism that could explain the effect of iron status on FGF23 production and cleavage. Iron deficiency may lead to (i) increased FGF23 transcription, through erythropoietin (EPO) or hypoxia‐inducible factor 1α (HIF1α) directly, and (ii) increased cleavage of FGF23, mediated by EPO.

The effect of iron status on FGF23 could be mediated by erythropoietin (EPO) and HIF1α (Fig. 1). EPO is upregulated in the setting of iron deficiency. It stimulates red blood cell production and reduces hepcidin to release iron from stores.^(^ ^12^ ^)^ It has been reported that EPO affects both production and cleavage of FGF23.^(^ ^13^ ^)^ In human studies with administration of recombinant EPO, iFGF23 remains normal or increases only slightly, while the increase in cFGF23 is more pronounced.^(^ ^13^ ^)^ HIF1α is stabilized in the setting of hypoxia or iron deficiency and activates the production of EPO. Interestingly, HIF1α may also affect FGF23 directly.^(^ ^8^ ^)^ David and colleagues conducted a study in mice who were injected with IL‐1β to mimic a pro‐inflammatory state, leading to functional iron deficiency. These mice were found to have increased cFGF23, while iFGF23 remained unchanged. When IL‐1β‐injected MC3T3‐E1 osteoblast‐like cells were pretreated with a HIF1α inhibitor, the effects of IL‐1β on *FGF23* mRNA expression and FGF23 protein were partially blocked, indicating that HIF1α may target FGF23 transcription directly.^(^ ^8^ ^)^

We acknowledge the limitation of our case study that the cFGF23 and iFGF23 assays used in our research lab are not meant for clinical practice. However, the iFGF23 concentration in our patient was comparable to the healthy control, and it is clear that our patient had a very low i:cFGF23 ratio, which can point to untreated iron deficiency.^(^ ^3^ ^)^ In iron deficiency, iFGF23 remains mostly unchanged, but it is possible that iFGF23 concentrations were increased at a certain point in time, causing the mild hypophosphatemia. However, other causes of mild hypophosphatemia should be considered, for example, hyperparathyroidism, use of alcohol, and less well‐known causes of hypophosphatemia such as obesity and Cushing's disease.^(^ ^1^, ^14^, ^15^ ^)^ After treatment of the iron deficiency, serum phosphate concentrations were normal, but the complaints of the patient only partly resolved. The muscle pain remained, which suggests that the mild hypophosphatemia was not clinically significant and that there is another cause for the complaints than hypophosphatemia. Still, her high cFGF23 concentrations led to extensive diagnostic procedures and a significant diagnostic delay, which would not have been conducted had we known iFGF23 concentrations. Alarmingly, the fact that cFGF23 does not necessarily reflect iFGF23 concentrations is not incorporated in commonly applied diagnostic algorithms of hypophosphatemia.^(^ ^6^ ^)^ In the Netherlands and also in other countries, hospitals only use cFGF23 assays and not iFGF23 assays, even though Hartley and colleagues found that measurement of iFGF23 is superior to cFGF23 in making a diagnosis of FGF23‐mediated hypophosphatemia.^(^ ^16^, ^17^, ^18^ ^)^

In conclusion, increased cFGF23 concentrations, especially in the setting of hypophosphatemia, can be wrongly classified as a FGF23‐associated disorder, while iFGF23 concentrations may be normal, for example in the setting of iron deficiency. Our case illustrates the limitation of cFGF23 assays, and we urge clinicians to be aware that cFGF23 concentrations do not necessarily reflect iFGF23 concentrations and that other causes should be considered, including iron deficiency as in our case. Another lesson we learned is that one should be cautious to attribute general complaints like fatigue and muscle weakness to a mildly decreased serum phosphate concentration, and keep an open mind to alternative diagnoses.

## Author Contributions

**Ariadne Bosman:** Conceptualization; formal analysis; investigation; visualization; writing – original draft; writing – review and editing. **Danielle M. A. Ratsma:** Conceptualization; formal analysis; investigation; writing – review and editing. **Bram C.J. van der Eerden:** Conceptualization; resources; supervision; writing – review and editing. **M. Carola Zillikens:** Conceptualization; resources; supervision; writing – review and editing.

## Disclosures

The authors have no conflicts of interest to declare.

### Peer Review

The peer review history for this article is available at https://www.webofscience.com/api/gateway/wos/peer-review/10.1002/jbm4.10790.
